# Advanced Characterization of Self-Fibrillating Cellulose Fibers and Their Use in Tunable Filters

**DOI:** 10.1021/acsami.1c06452

**Published:** 2021-06-09

**Authors:** Yunus Can Gorur, Michael S. Reid, Céline Montanari, Per Tomas Larsson, Per A. Larsson, Lars Wågberg

**Affiliations:** † Department of Fibre and Polymer Technology, KTH Royal Institute of Technology, Teknikringen 56, SE-100 44 Stockholm, Sweden; ‡ Wallenberg Wood Science Center, Teknikringen 56-58, SE-100 44 Stockholm, Sweden; § RISE Bioeconomy, Drottning Kristinas väg 61, P.O. Box 5604, SE-114 86 Stockholm, Sweden

**Keywords:** cellulose fibers, filter paper, CNF, nanofibrillation, green materials

## Abstract

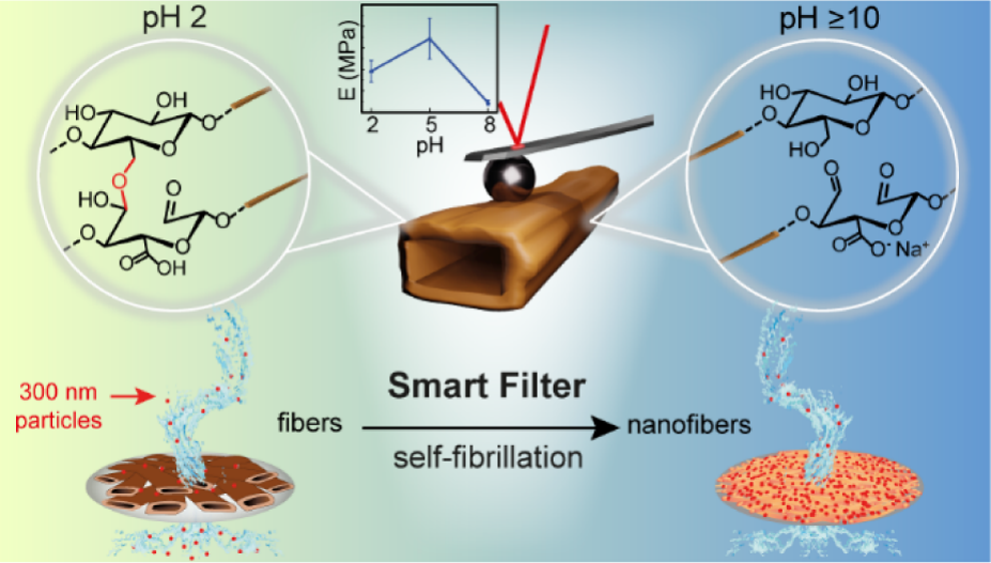

Thorough characterization and fundamental understanding of cellulose fibers can help us develop new, sustainable material streams and advanced functional materials. As an emerging nanomaterial, cellulose nanofibrils (CNFs) have high specific surface area and good mechanical properties; however, handling and processing challenges have limited their widespread use. This work reports an in-depth characterization of self-fibrillating cellulose fibers (SFFs) and their use in smart, responsive filters capable of regulating flow and retaining nanoscale particles. By combining direct and indirect characterization methods with polyelectrolyte swelling theories, it was shown that introduction of charges and decreased supramolecular order in the fiber wall were responsible for the exceptional swelling and nanofibrillation of SFFs. Different microscopy techniques were used to visualize the swelling of SFFs before, during, and after nanofibrillation. Through filtration and pH adjustment, smart filters prepared *via in situ* nanofibrillation showed an ability to regulate the flow rate through the filter and a capacity of retaining 95% of 300 nm (diameter) silica nanoparticles. This exceptionally rapid and efficient approach for making smart filters directly addresses the challenges associated with dewatering of CNFs and bridges the gap between science and technology, making the widespread use of CNFs in high-performance materials a not-so-distant reality.

## Introduction

To fully harness the potential of our sustainable resources, it is necessary to develop a deep understanding of their chemistry, structure, and behavior in different environments. However, unlike synthetic materials which, at their outset, can be designed at the nanometer level, the huge diversity of natural resources can make the extraction and thorough characterization of the different components of these materials challenging. Nonetheless, many investigations have led to significant advances of both bio-based and bio-inspired materials. An excellent example of this is the mussel foot protein, and specifically, the role of catechol binding in adhesion and cross-linking.^1^ Understanding the deployment, assembly, and properties of the protein residues has led to the development of many bio-inspired wet adhesives, composites, and self-healing materials. Similarly, wood-based cellulose fibers have been thoroughly investigated and chemically modified to produce an impressive variety of products ranging from paper and packaging to biomedical devices and electronics.^2−4^ However, as the need for sustainable materials continues to increase, so too does the need for new advanced processes and chemistries for the application of cellulose fibers. To this end, fiber wall swelling and the efficient production of nanocelluloses, in the form of cellulose nanocrystals and cellulose nanofibers (CNFs), are of significant interest as their use could potentially replace certain non-renewable and non-biodegradable materials.

To date, CNFs are largely obtained by applying mechanical shear to cellulose fibers *via* energy-intensive methods, such as grinding,^5^ microfluidization,^6^ and/or high-pressure homogenization,^7^ in order to liberate individual or groups of nanofibrils from the fiber wall. To improve the extraction efficiency, a variety of pretreatment procedures have been used to facilitate nanofibrillation, including enzymatic hydrolysis,^8^ carboxymethylation,^9^ 2,2,6,6-tetramethyl-1-piperidineyloxy (TEMPO) oxidation,^10^ sequential periodate-chlorite oxidation,^11^ and the use of peracetic acid for lignin removal while preserving the hemicellulose in the delignified fibers.^12^ In general, pretreatment facilitates nanofibrillation by either preferential oxidation and disruption of the cellulose network^13,14^ or by the introduction of charged groups that promote the buildup of osmotic pressure in the fiber wall.^15,16^ Recently, however, it has been shown that a combination of two pretreatment methods, in precise order, can yield self-fibrillating cellulose fibers (SFFs) that can be handled and processed as conventional cellulose fibers but can additionally be nanofibrillated on demand without the need for energy-intensive mechanical disintegration.^17^ This provides an excellent opportunity for CNFs to make an impact as a green material since these modified fibers can be processed using the conventional papermaking infrastructure and be nanofibrillated as needed before use, eliminating many of the challenges associated with dewatering. However, to take full advantage of this material, a thorough understanding of fiber swelling and nanofibrillation is needed.

Numerous methods have been used to establish a correlation between cellulose fiber properties and swelling behavior. Atomic force microscopy (AFM) nanoindentation can provide direct measurements of individual fibers in different environments,^18−21^ fiber saturation point (FSP) measurements in combination with charge measurements can be used to estimate the buildup of osmotic pressure inside the fiber wall,^22,23^ optical microscopy can be used to observe the swelling of fibers,^24^ and solid-state nuclear magnetic resonance (NMR) spectroscopy can be employed to evaluate structural changes within treated cellulose fiber walls.^25,26^ Pretreatment and fiber swelling play critical roles in nanofibrillation and have been the subject of numerous investigations.^13,15,16,27,28^ However, despite having well-studied pretreated fibers and their nanofibrillated counterparts, there is a lack of comprehensive literature characterizing the structural changes that occur within the fiber wall during swelling and nanofibrillation. Largely, this can be attributed to the energy-intensive mechanical processes required to facilitate nanofibrillation and the fact that it is technically challenging to analyze fiber structures *in situ* during these processes. As a result, nanofibrillation efficiency is often related to the number of passes required to achieve individualized CNFs or by the energy consumed.^5,15,29−31^ This result-oriented approach, while being practical, risks overlooking important swelling phenomena that could be of strategic importance to nanofibrillation of fibers to produce CNFs and their use in advanced applications, such as smart filtration.

Cellulose-rich fibers and papers made thereof have long been used as filters to remove both airborne and waterborne contaminants. Recently, nanocelluloses have been utilized in a variety of water remediation and separation applications both as adsorbents and membranes.^32−34^ Ion remediation utilizes the high specific surface area of nanocelluloses and the specific chemistry on the cellulose surface to bind mobile ions. Comparatively, particle filtration relies on size exclusion to remove particles such as debris and pathogens. Although coarse filters from cellulose fibers are commonplace, recent examples of nanocellulose membranes have shown that the narrow pore size of the membranes can effectively remove both bacteria and viruses from drinking water.^34^ Although these filters have shown great promise in a wide range of applications, limiting factors are the high opposing pressures and costly procedures that are required to retain CNFs during the formation of nanocellulose networks. As a result, it would be advantageous to rapidly produce nanocellulose membranes, capable of filtering sub-micron particles, using low cost, standard filter papers as a base structure.^35^

Here, we report a detailed investigation of the swelling and evolution of the fiber structure with respect to chemical treatments and pH by combining established direct and indirect fiber characterization methods and gel swelling theories. The swelling behavior of TEMPO- and TEMPO-periodate-oxidized cellulose fibers was determined by FSP and charge measurements, and these data were then used to model the swelling behavior of the fibers. AFM nanoindentation measurements were used to evaluate the elastic modulus of the fiber wall, and solid-state NMR was used to evaluate the structural changes of the fibrils within the fibers resulting from the chemical treatments. *In situ* fiber swelling was further visualized by cross-polarized optical microscopy (POM) to provide a necessary understanding of fiber stability during self-fibrillation. The information gained from the above investigations was then used to develop proof of concept of smart filters, capable of converting a conventional filter paper into a microfilter within seconds using vacuum filtration. Initial demonstrations show how these fibers could be used to make pH-responsive smart filters with flow regulation and nanoparticle retention capabilities with a simple pH adjustment.

## Materials and Methods

### Materials

Fully bleached, never-dried, softwood kraft pulp fibers (a mixture of Norwegian spruce and Scots pine) were obtained from BillerudKorsnäs AB (Gruvön pulp mill, Grums, Sweden). The fibers were industrially beaten (114 kW h/*t*) and had a water retention value of 2.0 g/g, measured according to a simplified version of the WRV SCAN 68:00.^25^ Sodium hypochlorite (10–15% solution), TEMPO (free radical), hydroxylamine hydrochloride, sodium bromide, sodium (meta)periodate (99%), 2-propanol (99.9%), tetraethyl orthosilicate (98%), dextran 2000, and ammonia solution (25%) were all purchased from Sigma-Aldrich and were used as received. Sodium hydroxide and hydrochloric acid standard solutions (1 M) were obtained from Merck Millipore.

### Modification of Cellulose Fibers

TEMPO oxidation of cellulose fibers was performed using the TEMPO/NaBr/NaClO method in water at pH 10.5.^10^ In short, 0.1 mmol TEMPO, 1 mmol NaBr, and 9.7 mmol NaClO per gram of dry fiber were added to a 12 g/L fiber suspension and allowed to react for 1.5 h under gentle stirring at room temperature. The reaction pH was maintained at 10.5 by dropwise addition of 0.5 M NaOH to the suspension yielding TEMPO-oxidized cellulose fibers with a charge density of 1.2 mmol/g. SFFs were prepared by a further periodate oxidation of the TEMPO-oxidized fibers, according to a previously reported procedure.^17^ For the periodate oxidation, 3.0 g of sodium periodate was added per gram of dry TEMPO-oxidized fiber to a 12 g/L fiber suspension under gentle stirring. To limit side reactions, the fibers were oxidized for 24 h at room temperature in the dark, and 6.3 vol % 2-propanol was added to the suspension as a radical scavenger.^36^ Both reactions were terminated by filtration and thorough washing with deionized water.

### Chemical and Structural Characterization of Cellulose Fibers

The total charge of the fibers was determined *via* conductometric titration using a Metrohm 702 SM Titrino titrator, according to the SCAN-CM 65:02 standard. Each measurement was performed in triplicate. The aldehyde content was determined by titration with NaOH after reaction with hydroxylamine hydrochloride, which reacts with the aldehydes to release a stoichiometric amount of protons.^37^ Each measurement was performed in triplicate. Fourier transform infrared spectrometry (FTIR) of the modified fibers in their protonated forms was performed using a PerkinElmer Spectrum 100 FTIR equipped with a diamond attenuated total reflection crystal (Gaseby Specac Ltd, UK). The spectra were recorded at room temperature taking the average of eight scans with 4 cm^–1^ resolution in the range of 600–4000 cm^–1^. Fiber dimensions were measured optically using a L&W Fiber Tester Plus (Lorentzen & Wettre Products, Stockholm) from a sample pool of approximately 10,000 fibers per sample using the ISO 16065-2 standard.

### Solid-State NMR Spectroscopy

Solid-state cross-polarization magic angle spinning carbon-13 NMR spectroscopy (CP-MAS ^13^C NMR) spectra were obtained using a Bruker Avance III AQS 400 SB instrument operating at 9.4 T, fitted with a double air-bearing two-channel probe head. Samples were packed uniformly in a 4 mm zirconium oxide rotor. All measurements were performed at 296 (±1) K and pH 3.5. The MAS rate was 10 kHz. Acquisition was performed with a CP pulse sequence using a 2.95 μs proton 90° pulse, an 800 ms ramped (100–50%) falling contact pulse, and a 2.5 s delay between repetitions. A SPINAL64 pulse sequence was used for ^1^H decoupling. A Hartman–Hahn matching procedure was performed on glycine, and the chemical shift scale was calibrated to tetramethyl silane by assigning the data point of maximum intensity in a alfa-glycine carbonyl signal, a chemical shift of 176.03 ppm. Lateral fibril dimensions (LFDs) and crystallinity were evaluated by utilizing the quantitative nature of the C4 signal intensities. To do this, a simple model consisting of a fibril with a square cross-section was employed, and a conversion factor of 0.55 nm per cellulose polymer was used to calculate the fibril dimensions. The degree of crystallinity was evaluated by non-linear least-squares fitting of the C4 region in the NMR spectra.^38,39^

### Fiber Saturation Point

The FSP is defined as the amount of water contained within the water-saturated fiber wall, and it was assessed based on a method introduced by Stone and Scallan.^40^ The FSP method is a solute exclusion method based on the dilution of a dextran solution of known concentration after being mixed with fibers containing a carefully determined amount of water. The molecular mass of the dextran was chosen as high as possible so that the dextran molecules cannot enter the fiber wall pores of the pulp fibers. When the wet fibers are immersed in dilute aqueous solutions of dextran, the water trapped in the fiber wall pores cannot contribute to the dilution of the dextran solution. Hence, by measuring the change in the concentration of dextran after immersion of a known amount of pulp fibers, the FSP can be calculated. Here, a known amount of pulp with a previously measured dry content was placed in a small weighing bottle to which 1% dextran 2000 solution was added until the fibers were fully covered. The mixture was allowed to stand for 1–3 days with periodic shaking, after which the solution was withdrawn using a syringe. Using a polarimeter (Schmidt + Haensch GmbH & Co., Berlin, Germany), the final concentration of dextran was determined to calculate the FSP from the level of dilution. The measurements were repeated with 0.5 and 1.5% dextran solutions, and all measurements were performed in triplicate.

### Atomic Force Microscopy

Force measurements were collected using a MultiMode III (Veeco Instruments, Santa Barbara, USA) with PicoForce extension. Tipless AFM cantilevers (CCS27/tipless/noAl MikroMasch, Wetzlar, Germany), having a width of 30 μm and length of 100 μm, and a nominal spring constant of 1 N/m were calibrated^41^ and used for all experiments. Spherical silicon dioxide particles (Duke Scientific, Palo Alto, USA) with an average radius of 5 μm were mounted to the end of the cantilever using thermal glue. Cellulose fibers were drop-casted on clean^42^ silica wafer surfaces from a 0.1 g/L fiber suspension. All indentation measurements were performed with the aid of an AFM liquid cell containing 10 mM NaCl to account for the Donnan effect, unless otherwise stated. Fibers were allowed to equilibrate for a minimum of 15 min following the change in pH within the cell. Three force curves were collected at six different locations on two separate fibers for each sample. Force curves were collected with a tip velocity of 160 nm/s and a measurement depth of 100 nm. The results were evaluated and fitted to a Hertz model with a Poisson ratio of 0.23 using AFM Force IT v3 (ForceIT, Järna, Sweden) software to estimate the wet modulus of the fiber wall.^21,43^ AFM images were collected using a MultiMode 8 (Bruker, Santa Barbara, CA) in TappingMode with RTESPA-300 cantilevers having a resonant frequency of 300 kHz and spring constant of 40 N/m. SFFs were dispersed in water of different pH values and stirred with a magnetic stirrer at 1000 rpm for 5 min. Samples were spin-coated onto clean silica wafers pretreated with poly(allylamine hydrochloride).

### Polarized Optical Microscopy

Cellulose fiber swelling was visualized using a ZEISS Standard 25 ICS microscope equipped with cross polarizers. Cellulose fibers were drop-cast onto glass slides and placed in a custom built liquid cell in which the bulk pH was regulated using a peristaltic pump. Images were collected at 30 s intervals as the pH was slowly increased from 2 to 10 over a period of 10 min.

### Preparation and Testing of SFF Smart Filters

A 1 g/L SFF suspension containing 10 mM NaCl was brought to pH 2 and mixed for 5 min to ensure homogeneity. Using a vacuum filtration setup with 20 mm radius, a 40 μm thick layer of SFFs was formed by filtering the SFFs through a Whatman grade 4 filter paper (85 g/m^2^) to obtain a double-layered structure with a potential to be converted to a nanofilter following the self-fibrillation procedure. The flow rate through the SFF filters was measured at different pH values by monitoring the time needed to filter 50 mL distilled water with pH values ranging from 2 to 12. Filtration experiments were performed at 22 °C and under ambient conditions, while the vacuum pressure was measured as −0.9 bar using a manometer.

Nanoparticle filtration was examined by using model silica particles. Silica particles with an average diameter of 315 nm were prepared following a modified Stöber process.^44^ Briefly, 360 mL of ethanol and 70 mL of ammonia solution were stirred vigorously for 20 min. A solution containing 7 mL of tetraethyl orthosilicate and 28 mL of ethanol was quickly added to the ethanol and ammonia solution and allowed to react for 2 h at room temperature under vigorous stirring. The particles were then centrifuged at 4000*g* for 10 min and washed four times with distilled water. Nanoparticle filtration was examined by filtering a 10 mL (0.03 wt %) dispersion of silica particles through the SFF filters at pH 2 and 12. The filtrate was collected and oven dried in order to calculate silica particle retention in the smart filter.

### Scanning Electron Microscopy

The surface morphologies of the unfibrillated (pH 2) and fibrillated (pH 12) fibers and smart filters made thereof were studied using a field-emission scanning electron microscope S-4800 (Hitachi, Japan). Prior to imaging, all specimens were exchanged to ethanol and CO_2_ supercritical dried (Autosamdri-815, Tousimis, USA) and sputter-coated for 20 s with Pt/Pd (Cressington R208, UK).

X-ray energy dispersive spectroscopy (EDS) analysis was carried out to determine the silica particle distribution in the smart filters using scanning electron microscopy (SEM) at an accelerating voltage of 7 kV equipped with an X-Max 80 mm^2^ detector (Oxford Instruments, UK).

## Results and Discussion

### Changes in Fiber Structures as a Result of Chemical Modification

**Figure 1:**
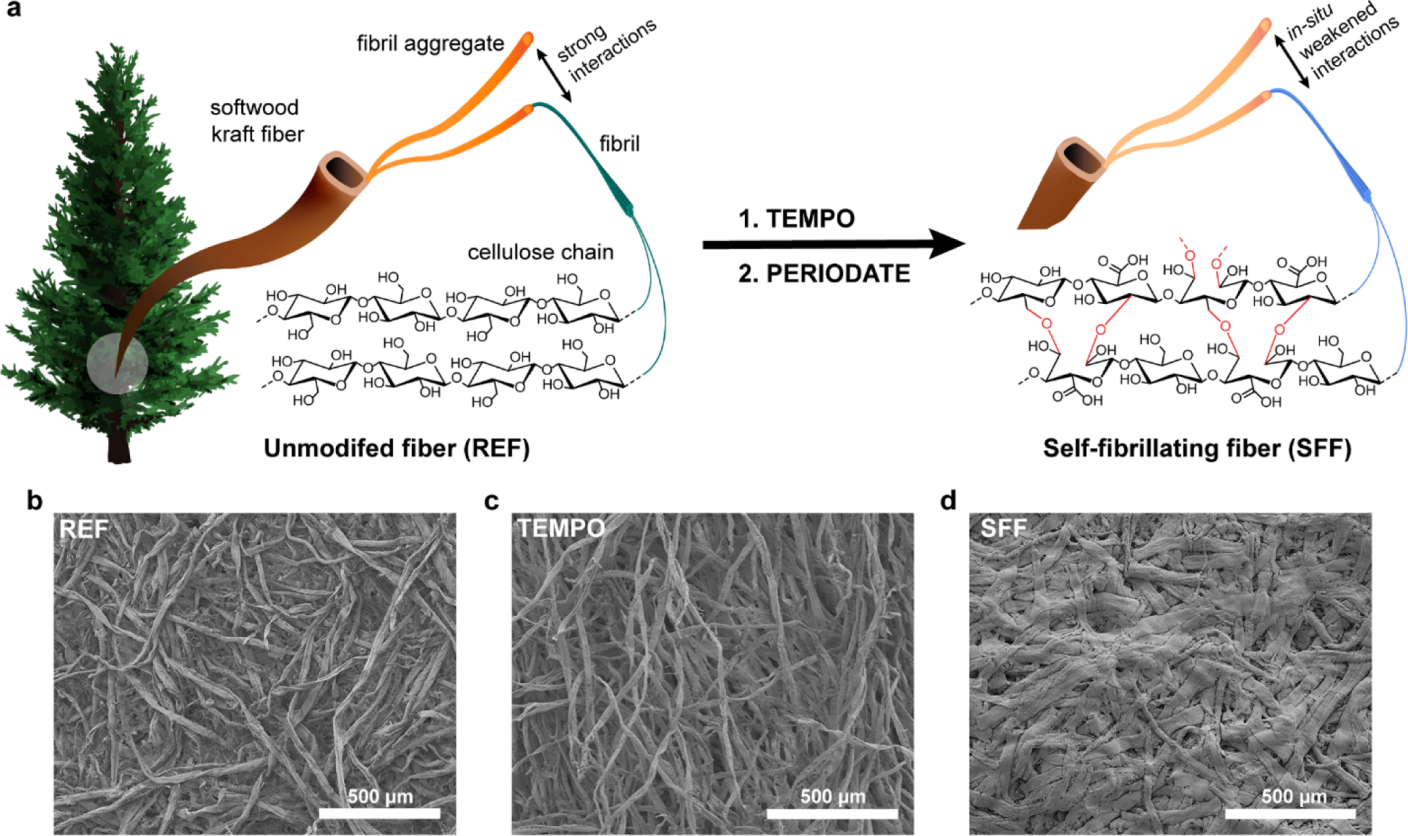
(a) Schematic description of how the sequential TEMPO and periodate oxidation are used to obtain SFFs (at low pH) with a built-in self-fibrillation functionality. Morphology of the (b) unmodified fibers (REF), (c) TEMPO-oxidized fibers (TEMPO), and (d) TEMPO-periodate-oxidized SFFs at pH 2 under SEM.

A sequential TEMPO-periodate oxidation (Figure 1a) of never-dried softwood kraft pulp fibers was used to produce SFFs. Previous work has demonstrated that the initial TEMPO oxidation introduces carboxyl groups that facilitates swelling without completely liberating the fibrils, whereas the subsequent periodate oxidation introduces aldehydes by opening the C2–C3 positions in the anhydroglucose unit along with oxidizing the outer layer of the cellulose fibrils, thereby altering the supramolecular structure of the fiber wall, making nanofibrillation upon swelling possible.^17^ Prior to chemical modification, the unmodified reference fibers (REF) had a total charge density of 0.1 mmol/g and aldehyde content of 0.0 mmol/g. Following TEMPO oxidation, the total charge density and aldehyde content of the TEMPO-oxidized fibers (TEMPO) increased to 1.2 and 0.2 mmol/g, respectively. The TEMPO fibers were subsequently periodate oxidized, resulting in SFFs with a charge density and aldehyde content of approximately 0.8 and 3.2 mmol/g, respectively. TEMPO oxidation had a yield of more than 90%, whereas the subsequent periodate oxidation had a yield of approximately 85%. It should also be noted that a considerable amount of material loss occurred during the transfer of the materials while washing the excess chemicals. The evolution of the measured chemical properties of the fibers is summarized in Table 1 where TEMPO oxidation increases the total charge density of the fibers as it introduces carboxyl groups to the C6 position of the anhydroglucose unit.^10^ The subsequent periodate oxidation introduces aldehydes by opening the anhydroglucose unit in the C2–C3 position, thus increasing the aldehyde content within the fibers. The observed reduction of charge following the periodate oxidation is presumed to be due to loss of highly charged cellulose chains *via* peeling from the modified cellulose fibril surfaces.^17,45−47^ FTIR spectra of the TEMPO-oxidized and TEMPO-periodate-oxidized samples show similar changes to the total charge density and aldehyde content of the fibers, further supporting the hypothesis of peeling of highly charged glucan polymers from the fibril surfaces inside the fiber wall (Figure S1). The overall structure of the macroscopic cellulose fibers remains relatively unchanged following both TEMPO and periodate oxidations (Figure 1b–d). The TEMPO-oxidized fibers appear more twisted and curled along the fiber axis with a higher concentration of surface debris compared to the reference fibers, whereas TEMPO-periodate-treated fibers have a much smoother appearance compared to both the reference and the TEMPO-oxidized fibers. Analysis of fiber dimensions shows that following TEMPO-periodate modification, the length of the fiber is slightly reduced and the width of the fiber increases due to the weakening of the fiber wall (Table S1). Nonetheless, SFFs can be handled and utilized using conventional paper making processes without the massive challenges of long dewatering times and high water retention values associated with the high specific surface area of the CNFs.

**Table 1:** Charge Density and Aldehyde Content of the Fibers after the Chemical Modifications

sample	charge density (mmol/g)	aldehyde content (mmol/g)
REF	0.1	0.0
TEMPO	1.2	0.2
SFF	0.8	3.2

**Figure 2:**
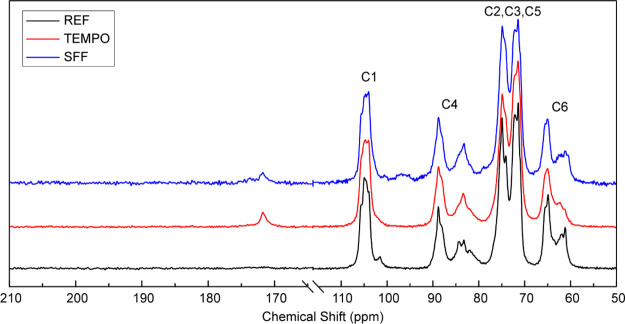
Solid-state CP-MAS ^13^C NMR spectra for the REF, TEMPO, and SFFs. The samples were never-dried, and the measurements were made at pH 3.5.

The supramolecular structure of the modified and unmodified cellulose fibers was examined in the wet state using solid-state CP-MAS ^13^C NMR. Figure 2 shows characteristic carbon-13 signals for cellulose I in the 50–110 ppm region where the changes in peak intensity show modification of the supramolecular structure of the cellulose fiber surface. The changes in intensity are further supported by the integral values of the carbon-13 peaks (Supporting Information, Tables S2–S4). Specifically, a multiplet at 105 ppm assigned to the C1 carbon of cellulose, two resonance signals near 88 and 85 ppm corresponding, respectively, to the C4 carbon in ordered and disordered fibril surface regions, and a similar splitting of the signals exist in the signals originating from the C6 carbons near 65 and 62 ppm, also corresponding to the ordered and disordered regions, respectively.^48,49^

TEMPO-oxidized cellulose shows a decrease in the carbon signal intensity between 61 and 63 ppm, corresponding to the modification of the C6 primary hydroxyl, which is in accordance with the well-known selectivity of TEMPO oxidation.^16,50^ Similarly, the appearance of the carbon signal associated with the C6 carboxyl groups at 172 ppm and the decrease in signal intensity near 101 ppm, resulting from the ionization and dissolution of the hemicelluloses, can also be seen following TEMPO oxidation.^49^ Analysis of the C4 region of the TEMPO spectra suggests that the surface of the cellulose fibrils was significantly altered due to the oxidation.^38^ Specifically, a slight broadening and a redistribution of signal intensity upfield (toward lower chemical shift values) in the 82–86 ppm region are also indicative of a significant cellulose fibril surface modification. At a charge density of 1.2 mmol/g, assuming a cellulose fibril of 4 nm width, an estimate of the degree of fibril surface oxidation is circa 70%. This means that approximately three out of four hydroxymethyl groups were oxidized in the fibril surface polymers. The spectral changes observed in combination with the charge density (Table 1) suggests that the TEMPO oxidation disassembles fibril aggregates within the fiber wall, with an effective increase in the specific surface area of the fiber wall as a result, albeit the macroscopic fibers remain largely intact.^25^ Despite the changes to the fibril surfaces and the disordered regions, TEMPO oxidation is shown, as expected, to be largely a surface modification, and estimates of fibril dimensions and crystallinity do not differ significantly to those of the unmodified fibers (Table 2). In this respect, it is suggested that the introduction of charges *via* TEMPO oxidation begins to separate the fibrils within the fibril aggregates but not to the extent that ion-induced swelling alone can overcome the restraining forces of the fiber wall.^25,51^ Furthermore, it should be added that the NMR measurements had to be performed at low pH with the carboxyl groups in their protonated form, which means that the separation of the fibrils will be higher at higher pH when all the carboxyl groups are ionized.^25^

**Table 2:** LFDs Along with Crystallinity of the Modified and Unmodified Fibers Based on Spectral Fitting^a^

sample	crystallinity (%)	LFD (nm)
REF	57 (1)	4.7 (0.1)
TEMPO	56 (2)	4.6 (0.1)
SFF	58 (1)	4.8 (0.1)

a Standard errors are presented in parentheses.

The SFF spectrum exhibits similar characteristic signals as TEMPO-oxidized fibers along with changes in the signal intensities due to the subsequent periodate oxidation. A decrease in the carbon signal associated with the carboxyl groups at 172 ppm upon periodate oxidation is in good agreement with both the charge density measurements and the FTIR measurements as well as previously reported results.^45,46,52^ Additionally, the signal corresponding to the C6 primary hydroxyl groups on the fibril surface at 63 ppm, which was decreased upon TEMPO oxidation, increases following periodate oxidation. This is potentially due to dissolution of highly modified polymers, thereby exposing new surfaces with pristine primary hydroxyls. Similarly, a slight signal broadening observed near 105 ppm can be attributed to minor alterations to the supramolecular structure of the interior regions of the ordered cellulose I domains. As periodate oxidation acts first on the surface of the fibril aggregates, the modification then slowly proceeds to the interior of the aggregates and the fibrils, likely forming a core-shell-like structure.^14,53^ While minor disruption of the interior cellulose I structure can be observed, the 25% degree of periodate oxidation leaves the fiber wall largely intact with the average crystallinity remaining unchanged following periodate oxidation.^38,39^ These levels of oxidation have been shown to be mostly limited to the surface, and therefore, the resonance signal at 105 ppm remains largely unaffected with only minor broadening.^54^ Despite having an aldehyde content of 3.2 mmol/g, the SFFs have a distinct lack of resonance signals within the carbonyl region of 175–210 ppm. This indicates that the aldehydes, once formed, most probably rapidly recombine into intra- or interchain hemiacetal cross-links with the remaining hydroxyl groups, rather than existing in their free form.^14,49,53,55^ Hemiacetal moieties are evident by the broad signal in the region of 90–100 ppm, which is not present in the unmodified or TEMPO-oxidized fibers. It should be noted that the SFF spectrum does not show full agreement with the pronounced differences observed for periodate-oxidized celluloses at similar modification levels;^26,49^ this can be attributed to the fact that these studies use uncharged microfibrillated cellulose, whereas TEMPO-oxidized macroscopic cellulose fibers are used in the current work. These chemical and morphological differences between the starting materials (macroscopic fibers *vs* microfibrillated cellulose) presumably led to differences in the homogeneity of the periodate oxidation along with reflections of these differences on the respective NMR spectra.

Furthermore, while the presence of specific signals within the NMR spectra provide insights into the chemical structure of the fibrils, spectral fitting can provide estimates of the average LFD. Following both TEMPO and periodate oxidation, the LFDs do not change significantly, further reinforcing the insights that the modification is mostly limited to the surfaces of the fibrils.

**Figure 3:**
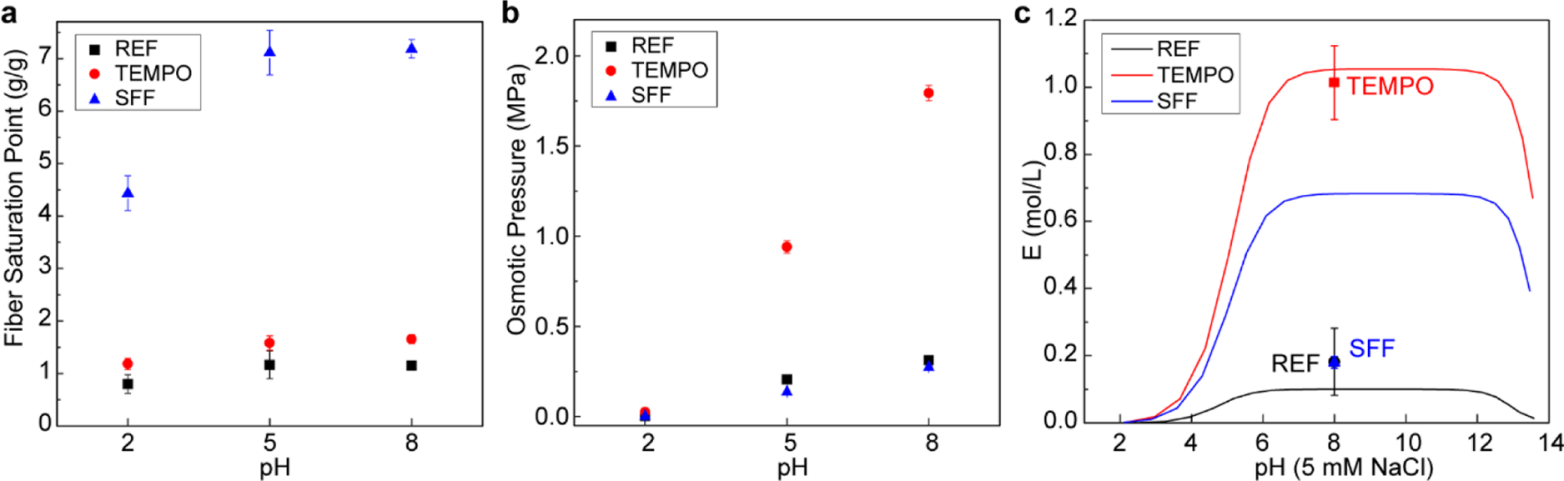
(a) FSP of the fibers measured at different pH values. (b) Osmotic pressure inside the fiber wall at different pH values calculated using Donnan theory. (c) Calculated swelling potential for the fibers and the experimental data obtained from the FSP measurements compared with each other to determine how well the theory describes the observed ion induced swelling of the fiber wall (the experimental points are placed at pH 8 where a plateau level in the FSP was attained).

The FSP results for the unmodified and modified fibers provide further insights into swelling behavior with respect to chemical modification and pH and allow these phenomena to be described as the balance between the three different osmotic pressure terms; the ionic contribution (π_ion_), the gel-solvent mixing contribution (π_mix_), and the network pressure contribution (π_def_), all of which make up the total swelling pressure (π_tot_) in the fibrillar network.^56^ The net balance of these contributing pressures determines the swelling of the fiber wall at equilibrium.^57,58^ Chemical modifications performed with the fibers can shift this equilibrium, by affecting the contributing factors, to the point where swelling forces may overcome the opposing restraining network forces with a small external stimuli. Swelling trends obtained from FSP measurements for all fibers were consistent with representative swelling theories;^59^ however, there are notable changes due to the significant swelling of SFFs following periodate oxidation (Figure 3).^23,25^

The FSP results show an increased swelling and water holding capacity of the fiber wall with increasing pH and oxidation levels (Figure 3a). Introduction of charges *via* TEMPO oxidation increases the FSP of the fibers. This increase in π_ion_, that is, the increase in the osmotic pressure due to the introduced charges, can also be calculated using the van’t Hoff equation as applied to Donnan theory (see the Supporting Information) particularly at pH > 4 where carboxyl groups start to become deprotonated (Figure 3b).^56−58,60^ Subsequent periodate oxidation, while reducing the overall charge, results in the most swollen pulp with SFFs showing nearly four times the FSP than TEMPO-oxidized fibers. While SFFs have a reduced π_ion_ due to the periodate modification (since the charge of these fibers is reduced), it can be concluded that the restraining π_def_, that is, the fiber wall integrity, is hence significantly reduced, thereby allowing more water into the fiber wall. This hypothesis is based on the assumption that it is not reasonable to assume that the π_mix_ is dramatically affected by the introduction of the dialdehyde groups. However, the quantitative influence of the periodate oxidation on the term π_mix_ is still to be quantified. Nevertheless, the reduction of both π_ion_ and π_def_ is evident in the calculated osmotic pressure whereby SFFs show a significant decrease compared to TEMPO fibers. This reduction is, as shown by NMR measurements, attributed to the decrease in the ordering of cellulose in the SFFs.

To further investigate the osmotic pressure and swelling within the fiber wall, the theoretical swelling potential (*E*), based on Donnan theory, was compared to the experimental data (see the Supporting Information for a detailed description of the calculations) (Figure 3c).^56^ Using the measured charge values for the different pulp fibers, the theoretical swelling potential curves were plotted using the definitions of mobile ion distribution (λ) and swelling potential. These curves were then compared with the experimental values obtained using the actual volumes, as determined from the FSP measurements in order to determine how well the theory describes the observed ion induced swelling of the fiber wall. The swelling potential is based on the excess concentration of ions within the fiber network compared to the concentration in the bulk water. The amount of excess ions, which is the most significant factor contributing to osmotic pressure buildup (π_ion_), depends both on the presence and dissociation of the carboxyl groups. Thus, the amount of excess ions is affected by pH and ionic strength. The excess ion concentration inside the fiber wall creates an osmotic pressure gradient, causing water to flow into the network to decrease this imbalance in the concentration. The resulting swelling and expansion of the fiber wall continue until the network pressure of the fiber wall constituents equals the osmotic pressure *via* resistance to deformation, π_def_. Comparing the predicted swelling potential to the measured values shows good agreement for REF and TEMPO fibers. SFFs, on the other hand, show an experimental result that was significantly lower than the predicted values. This difference is largely attributed to the disruption of the ordered cellulose in the fiber wall due to the periodate oxidation and the increased porosity within the fiber wall following this treatment. Specifically, FSP measurements show that dextran, with a hydrodynamic radius of 96 nm, was even able to penetrate SFFs, indicating that large pores are present within the fiber wall. As a result, Donnan theory, which inherently requires pores to be of similar or smaller size than the Debye length,^61^ overestimates the swelling pressure within the SFF network. The increased water holding capacity and fiber wall porosity of SFFs, while difficult to model and predict using conventional theories, have immense application potential as the large pores provide pathways for polymers and particles to enter and modify the interior of the fiber wall. On the other hand, while the FSP results support a decrease in the supramolecular order due to periodate oxidation, a similar expected decrease is absent from the crystallinity data (Table 2). This seemingly puzzling observation can presumably be explained by the nature and reaction mechanism of periodate oxidation where there are two rate constants, the faster of which preferentially takes place in the amorphous regions and then advances toward the crystalline regions.^62,63^ These heterogeneous oxidation conditions of cellulose may result in non-uniform swelling, which could explain the difference in experimental and theoretical swelling observed for SFFs. Furthermore, considering crystallinity calculations yield relative values and the measured spectrum almost always contains contributions from amorphous regions in the position of the crystalline peaks as well, crystallinity is often overestimated.^64^ All of these factors contribute to the apparent discrepancy between the crystallinity data and the inferences made from the FSP data concerning the supramolecular order in cellulose.

**Figure 4:**
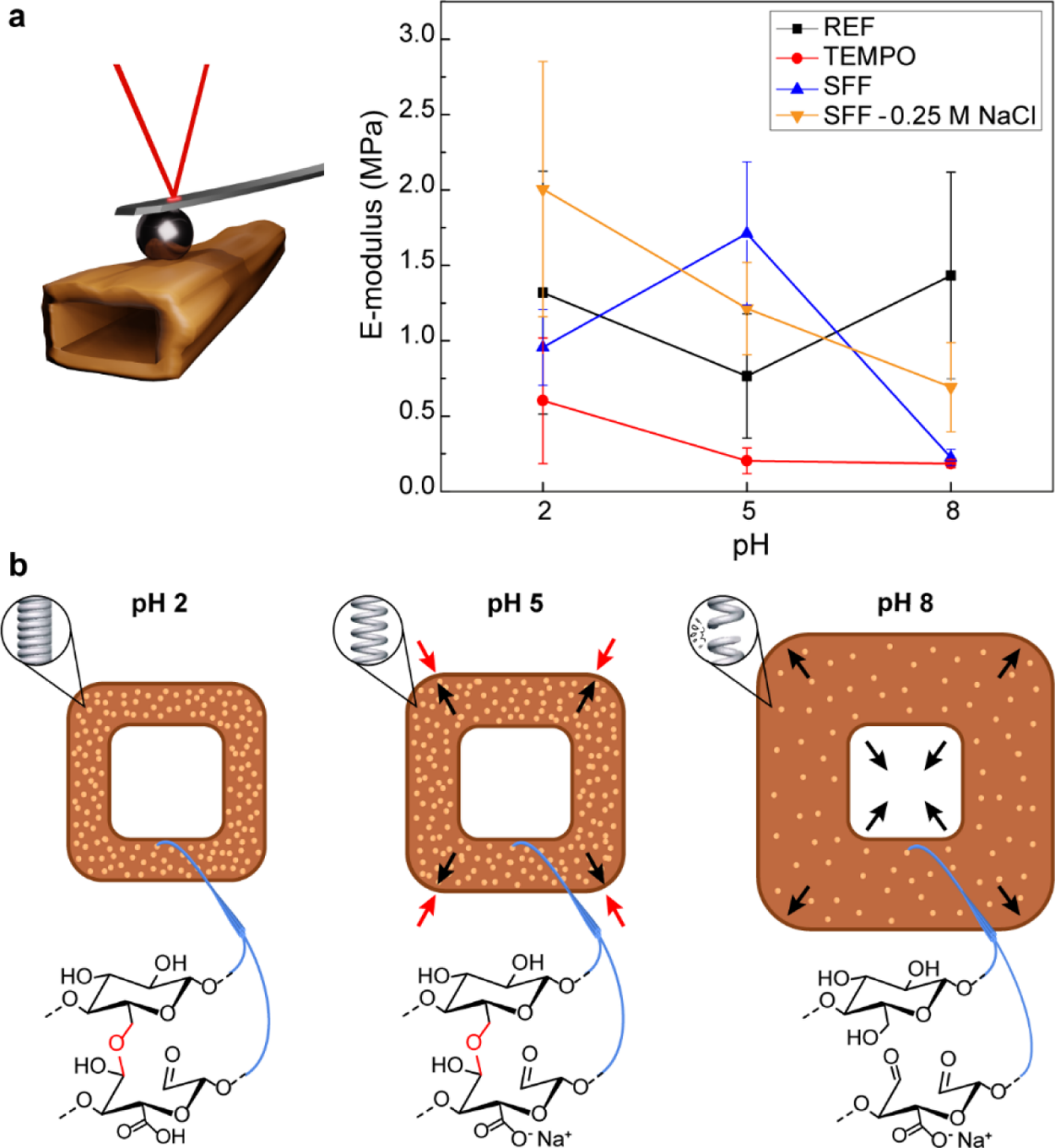
(a) Apparent *E*-modulus values for the fiber wall of modified and unmodified fibers obtained *via* AFM nanoindentation by fitting the data with the Hertz model to estimate the wet modulus of the fiber wall. (b) Scheme showing the presumed behavior of the fiber wall and the forces (π_def_ with red and π_ion_ with black arrows) acting on it at different pH values inside the fiber wall. In the inset circles, a spring is used as an analogy to represent the mechanical state of the fiber wall.

AFM nanoindentation was also used to probe the tangential apparent elastic modulus of the modified and unmodified fibers under increasing pH to further evaluate the effect of different treatments and to identify the mechanisms behind the self-fibrillation process (Figure 4a). For all fibers, the magnitude of the elastic modulus was in reasonable agreement with previously reported values despite the well-known heterogeneity of pulp fibers.^21,22,65^ As expected, the unmodified fibers did not show any significant variation in elastic modulus with respect to pH. This is attributed to the lack of charged groups that promote swelling, that is, the absence of charged native hemicelluloses.^12^ TEMPO fibers showed an overall reduced elastic modulus due to the swelling and softening of the fiber, resulting from the disruption of the supramolecular order in the cellulose and due to the introduction of charged groups in the fiber wall following modification. As the pH increases and the carboxyl groups become deprotonated, only a slight decrease in apparent elastic modulus is observed. Comparatively, SFFs show a distinct trend in which the apparent elastic modulus increases from pH 2 to pH 5, followed by a significant reduction at pH 8, matching that of TEMPO fibers. This unique behavior is attributed to the cross-linking hemiacetals in SFFs at pH < 8 and partial elimination of the cross-links at higher pH. Specifically, as the pH increases past the average p*K*_a_ of carboxylic acid groups the fiber swells, however, the hemiacetals restrain this expansion and the water filled fiber wall becomes more stiff, thus increasing the apparent elastic modulus of the fiber wall. As the pH is brought to 8, the cross-linking hemiacetals are beginning to break and the fiber swells nearly to the point of disintegration, lowering the apparent elastic modulus below the initial value at pH 2 (Figure 4b). Removing the effect of swelling due to the charged groups shows a distinctly different trend for SFFs as the pH increases. In 250 mM NaCl, where the carboxyl groups are effectively screened, the modulus continuously decreases as the pH increases, and the hemiacetals start to break. As a result, it is proposed that the combination of ion-associated swelling and the retention of the hemiacetal cross-links in SFFs are largely responsible for the increased tangential apparent elastic modulus at pH 5.

### Nanofibrillation

**Figure 5:**
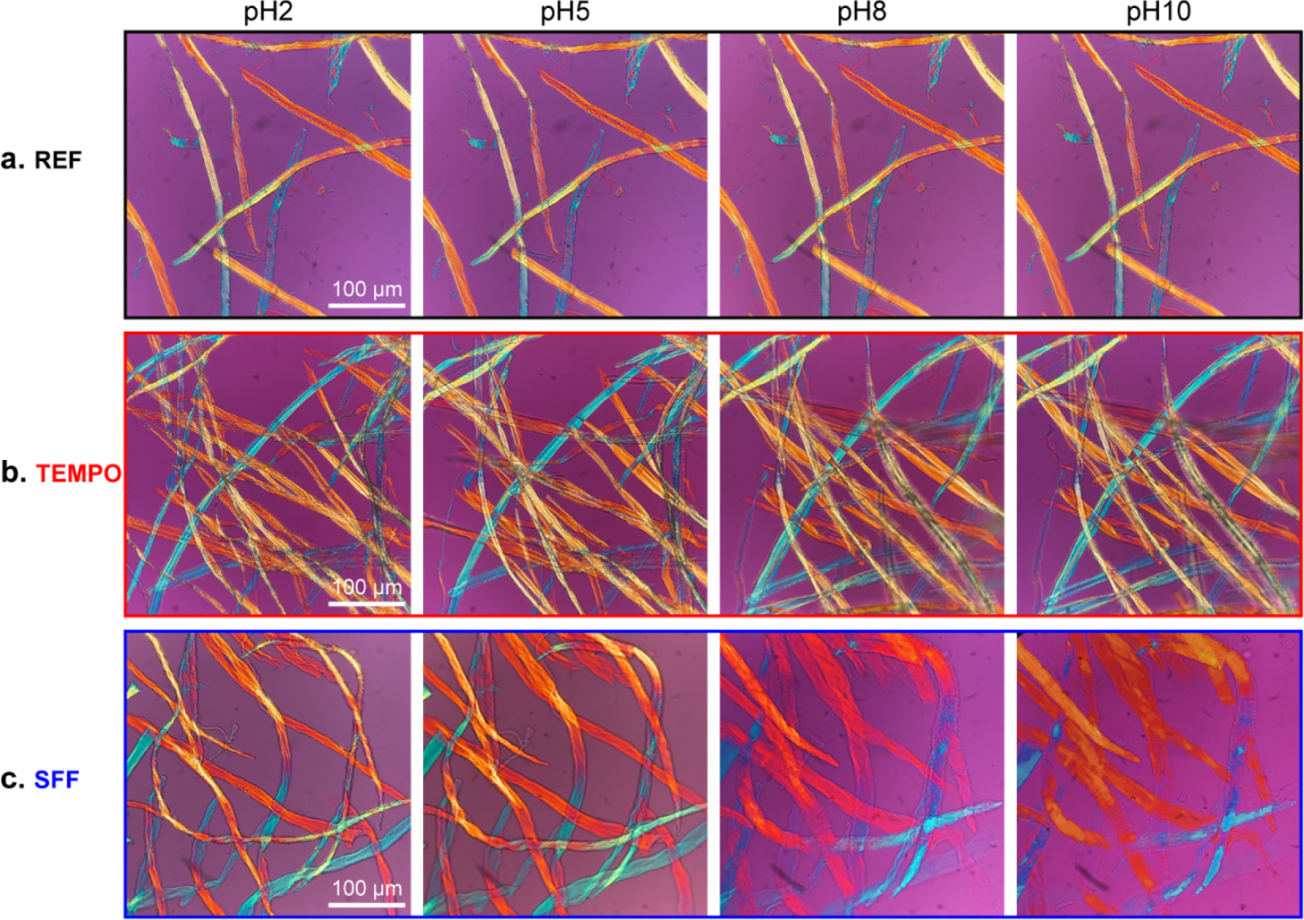
Cross-POM images of the modified and unmodified fibers obtained *in situ* at different pH values. (a–c) Image groups are obtained from REF, TEMPO, and SFF samples, respectively.

While a fundamental understanding of fiber swelling is of great importance, the self-fibrillation of cellulose fibers has the potential to significantly expand the utilization and production of high-value-added nanocellulose-based materials. POM images provide insights into the rapid self-fibrillation process, whereby SFF structures can be seen to significantly change by increasing from pH 2 to 10 in a matter of minutes (Figure 5) (see the Supporting Information for video). Minor swelling and “fuzzy” edges are evident in TEMPO fibers at pH 8 and 10, due to the highly charged fiber surface, and no significant changes are observed for unmodified fibers. Comparatively, noticeable swelling occurs within the SFFs at pH 5 and continues as the fiber diameter increases by nearly 10 times until almost complete fibrillation occurs at pH 10. It was observed that SFFs did not exhibit balloon-like structures during their swelling. Lack of balloon-like structures was attributed to the degradation of secondary layers in the fiber wall during the oxidations to the extent that the nanofibril confinement caused by swelling was removed.^24^ There is a significant increase in the transparency of the SFFs as the pH is increased, presumably due to the “loss” of cellulose in the form of CNFs on account of nanofibrillation. Surprisingly, despite a reduced impact from the hemiacetal cross-links and the strong electrostatic repulsion from the highly charged cellulose chains, some fibers retain their fiber-like structure. This is potentially due to strong van der Waals interactions within the ordered cellulose structure, the heterogeneous nature of the periodate oxidation, and the lack of mechanical agitation within the liquid cell.^31,66,67^

**Figure 6:**
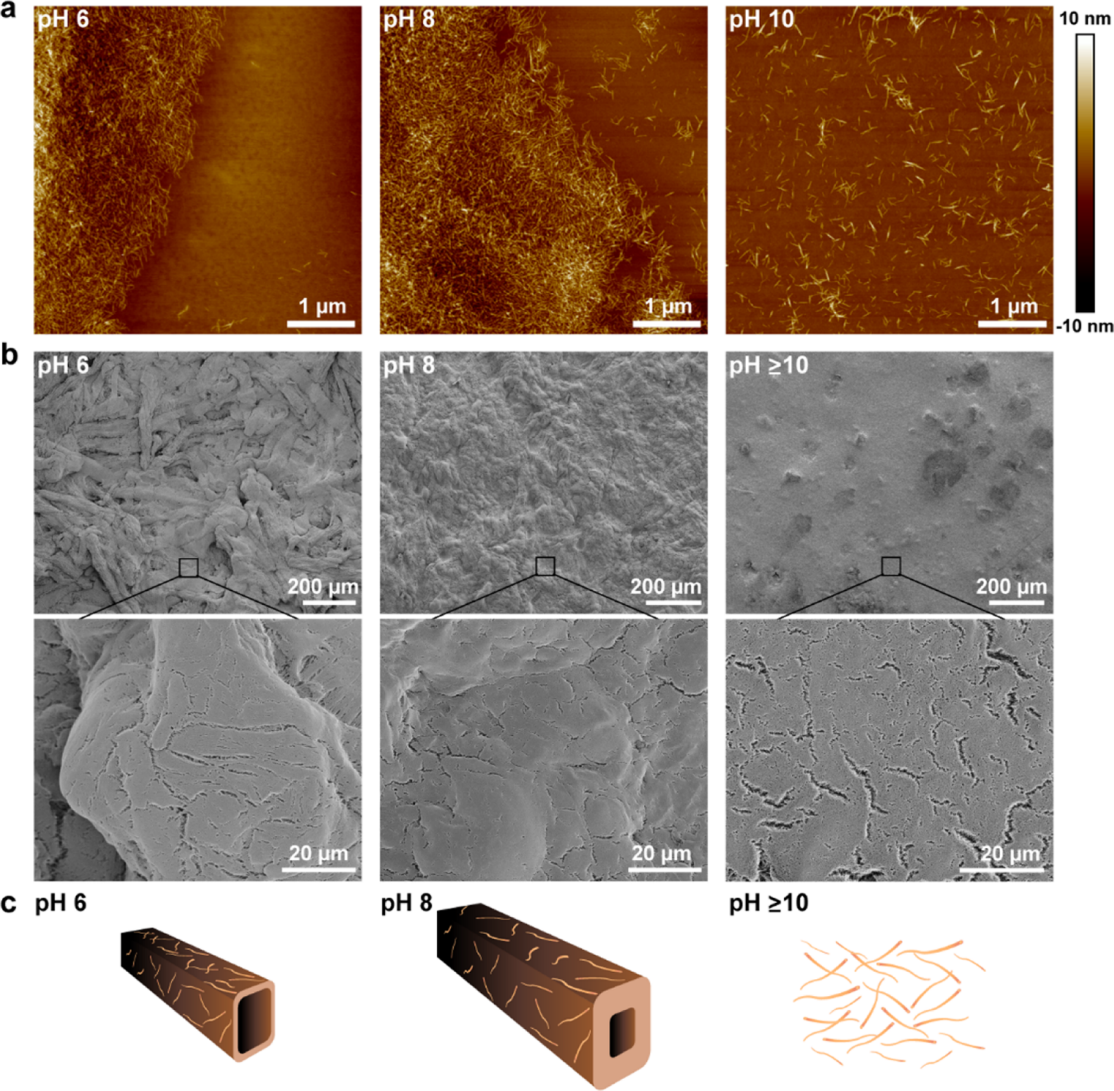
(a) AFM images of SFFs at pH 6, 8, and 10. (b) SEM images of SFFs at pH 6, 8, and ≥ 10. (c) Scheme depicting the swelling and nanofibrillation of SFFs upon a pH increase.

The nanofibrillation of SFFs is more apparent *via* AFM and SEM in which individual CNFs can be imaged. Figure 6a shows AFM images of SFFs following exposure to pH 6, 8, and 10. At pH 6 and 8, macroscopic fibers were present, and imaging the edge of these fibers reveals a fibrillar structure, held together, presumably, by cross-linking hemiacetals. Upon increasing the pH to 10 under gentle agitation and consequently removing hemiacetals, no evidence of macroscopic fibers could be seen, indicating that fibrillation of the fibers has occurred. This suggests that only a minor mechanical agitation is sufficient to completely individualize nanocellulose from the fiber wall at this pH. Similarly, SEM images (Figure 6b) show that for pH < 8, the fiber structure of SFFs is largely maintained; however, upon exposure to pH 10, self-fibrillation starts to occur, forming a highly porous interconnected network of nanocellulose (Figure 6c). This rapid transition from macroscopic fibers to nanocellulose opens numerous potential applications for SFFs, whereby the fibers can be handled and transported as conventional pulp fibers and fibrillated as needed to provide nanoscale structure and surface area.

### Preparation and Evaluation of Smart Filters

**Figure 7:**
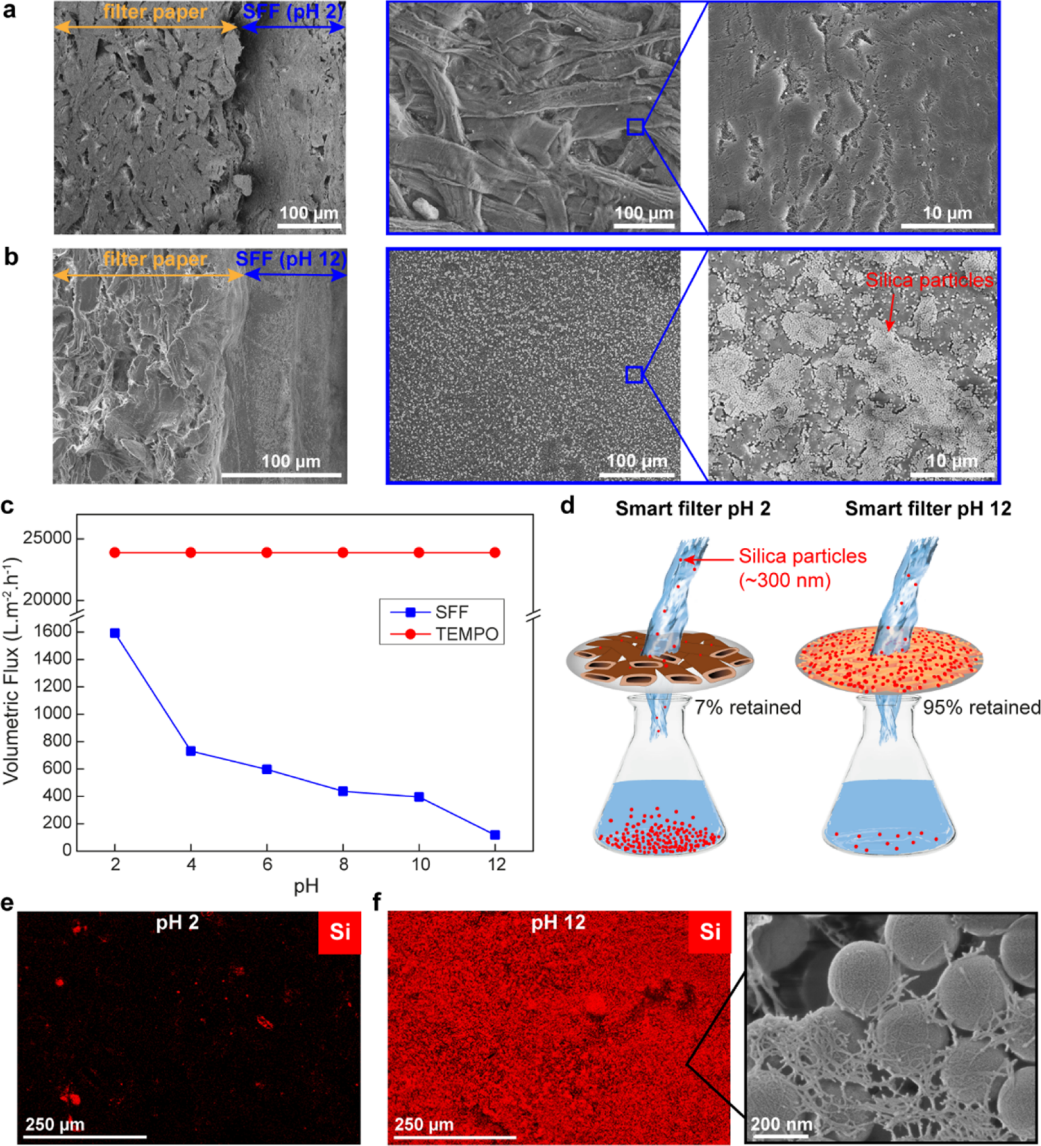
Cross-sectional and top-view images of smart filters (a) at pH 2 and (b) at pH 12. (c) Volumetric flux of water through the SFF smart filter (blue) and TEMPO fibers (red) at different pH values. (d) Schematic description of how the SFF smart filters change their morphology upon increasing pH and regulate flow and filtration of silica particles. (e) EDS images of the smart filters at pH 2 and (f) at pH 12 after filtration (Si is shown in red). The inset in (f) shows the high-resolution SEM image of silica particles buried in the nanofibril network of SFF smart filters.

A controllable nanoscale structure is critical for many filtering and separation applications. Submicron pore sizes that allow high flow rates while retaining pathogens, such as viruses and bacteria, are ideal for waste water treatment.^68^ Nanocellulose has been shown to be an effective material for filtration; however, the formation of nanocellulose-based filters often requires specialized membranes onto which nanocellulose is retained in order to create a “stand-alone” membrane.^69^ Moreover, current nanocellulose membranes, while being highly stable, are static and do not respond to the filtrate by changing the flow rate or structure. In contrast, SFFs can be handled as conventional fibers and respond rapidly to environmental changes. To demonstrate the potential use of SFFs in filtration applications, smart filters from SFFs were rapidly prepared by forming a thin layer of SFFs onto conventional Whatman filter papers (Figure 7). Due to the rather large dimensions of the unfibrillated SFFs (around 30 μm in diameter and >1 mm in length), these fibers can quickly be deposited and retained onto standard filter papers using conventional vacuum filtration. Large pores in the unfibrillated SFF network allow for deposition and dewatering of a >100 μm thick film to occur in a matter of seconds (Figure 7a). Upon increasing the pH to 12, the SFFs rapidly fibrillate, closing many of the large pores, resulting in a ∼40 μm thick interconnected CNF network on the filter paper (Figure 7b). The flow rate of the SFF filters can be easily tuned by a simple pH adjustment, with a 13-fold decrease by increasing the pH from 2 to 12 (Figure 7c). Moreover, the trend in the flow rate between pH 2 and 10 was reversible, possibly providing an opportunity to rapidly flush and cycle the membrane (Figure S3). This reversible behavior can presumably be explained by the ability of the partially or fully nanofibrillated SFFs to form aggregates, thanks to the recombination of hemiacetal linkages at low pH.^70^ As the filters are cycled, volumetric flux steadily decreases with increasing number of cycles, which is attributed to the accumulation of partially or fully nanofibrillated SFFs during the repeated swelling/deswelling sequences. This rapid change in the flow rate suggests that these smart filters can potentially be used as flow regulators in applications, for example, where a product pH needs to be maintained. Comparatively, TEMPO fibers show no change in the flow rate due to the lack of swelling and fibrillation of the fibers, as seen in the POM (Figure 5).

To examine the performance of SFF smart filters, 300 nm silica particles were used as the model particulate. The particles were filtered at pH 2 and 12 to investigate the particle retention of unfibrillated and fibrillated filters (Figure 7d). At pH 2, where SFFs are unfibrillated, only 7% of the silica particles were retained, whereas at pH 12, the same filter retained nearly 95% of the silica particles. SEM images of critical point dried filters show that while the surface of the unfibrillated SFFs have nanoscale structure, the large pores (>10 μm) between fibers allow silica particles to pass through (Figure S4). Comparatively, following fibrillation at pH 12, the large pores are closed and the filter densifies, thus facilitating the high retention of silica particles in the nanocellulose network. EDS images show small-localized clusters where silica particles are retained in the unfibrillated filter, whereas the fibrillated surface is completely covered by silica particles (Figure 7e,f). The significant increase in retention of the silica particles suggests that the extensive swelling of SFFs (approximately 10 times) is sufficient to close the majority of the micron-sized pores in the fiber network and with further optimization has the potential to retain more and even smaller particles.

## Conclusions

To effectively develop sustainable materials, a detailed understanding of the structure and chemistry of our natural resources is required. In this work, new insights into the swelling of TEMPO-oxidized and self-fibrillation of TEMPO-periodate-oxidized cellulose fibers were provided and used to rapidly prepare smart, responsive filters capable of retaining nanoscale particles. Using direct and indirect methods, a decreasing supramolecular order was found to occur in the fiber wall following chemical modifications, both of which contributed to the swelling and self-fibrillation of the modified cellulose fibers. However, TEMPO oxidation alone did not disrupt the cellulose I network within the fiber wall to sufficiently allow for self-fibrillation upon deprotonation of the carboxyl groups. Swelling and water retention of unmodified and TEMPO-oxidized fibers demonstrate the buildup of an osmotic pressure inside the fiber wall, which correlates well with Donnan and polyelectrolyte swelling theories. However, following periodate modification, large pores (>100 nm) present within the fiber wall limit the direct applicability of Donnan theory for SFFs. Hemiacetal cross-linking within SFFs was shown to help maintain the fiber structure at low pH and to provide unique mechanical properties under different pH values. Self-fibrillation of SFFs was observed to occur on the order of minutes at both the macro- and nanoscale with complete fibrillation occurring with only minor agitation. Utilizing the understanding of the fiber structure and swelling, the unique features of SFFs were used to form smart nanocellulose filters on conventional filter papers. The flow rate of the smart filters could reversibly be tuned by a simple pH adjustment and were capable of retaining nearly 95% of 300 nm nanoparticles. With further optimization, SFFs have the potential to more effectively retain nanoscale particles or act as flow regulators in a variety of applications.

## Supporting Information Available

FTIR curves of unmodified and modified fibers; fiber dimensions measured using a standard fiber analyzer; integrated signal intensities for modified and unmodified fibers; SEM and EDS images of the SFF smart filters; volumetric flux through the smart filters while the pH is cycled between pH 2 and 10; computation of the swelling potential using Donnan theory; and calculation of osmotic pressure inside the fiber wall (PDF)
Modified and unmodified fibers recorded *via* POM in the pH range of 2 to 12 showing the swelling and eventual nanofibrillation of the SFFs (MP4)
