# Supplementary material for: Advanced Characterization of Self-Fibrillating Cellulose Fibers and Their Use in Tunable Filters
Source: ACS Appl Mater Interfaces. 2021 Jun 9;13(27):32467–78. doi: 10.1021/acsami.1c06452 (PMC8289225; doi:10.1021/acsami.1c06452)
Supplement: Supplementary file 1 — am1c06452_si_001.pdf [file am1c06452_si_001.pdf]

Supporting Information

Advanced Characterization of Self-fibrillating Cellulose Fibers and their Use in Tunable Filters

Yunus Can Gorur,[†] *Michael Reid,**[,†] *Céline Montanari,*[†,‡] *Per Tomas Larsson,*[†,§] *Per A. Larsson,*[†] *and Lars Wågberg**[,†]

[†]Department of Fibre and Polymer Technology, KTH Royal Institute of Technology, Teknikringen 56, SE-100 44 Stockholm, Sweden

[‡]Wallenberg Wood Science Center, Teknikringen 56-58, SE-100 44 Stockholm, Sweden

[§]RISE Bioeconomy, Drottning Kristinas väg 61, Box 5604, SE-114 86 Stockholm, Sweden

*Corresponding author: mreid@kth.se, wagberg@kth.se

Supplementary FTIR Information

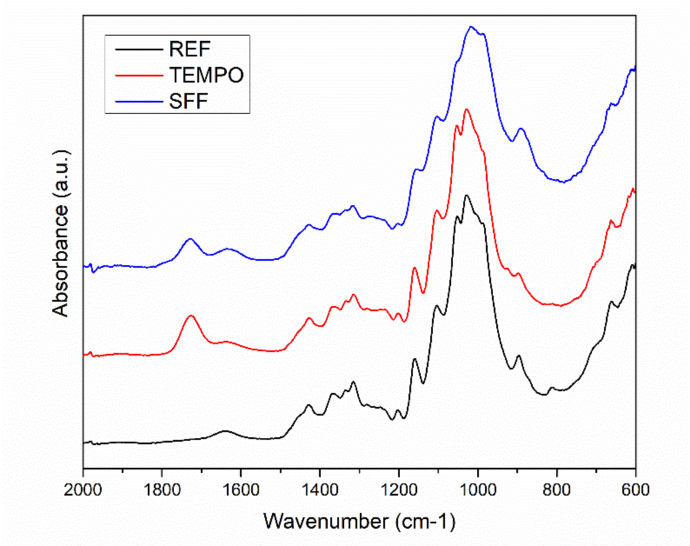

Figure S1. FTIR plot of unmodified and modified fibers

Supplementary Fiber Dimensions

Fiber dimensions were measured optically using a L&W Fiber Tester Plus (Lorentzen & Wettre Products, Stockholm) from a sample pool of approximately10000 fibers per sample using the ISO 16065-2 standard.

Table S1. Modified and unmodified fiber dimensions

	Mean Length (mm)	Mean Width (µm)	Mean fines (%)
**REF (ND SWKP)**	2.004	30.3	19.9
**SFF-H (unfibrillated)**	0.886	46.0	17.7
**SFF-Na (partially fibrillated)**	0.424	38.2	61.4

Supplementary NMR Information

Table S2. C6 integration regions of unmodified and modified fibers

C6 integration regions normalized to 1.0			**Before Oxidation(s)**	**After Oxidation(s)**
**Relative Surface Signal Intensities**	**REF**	0.36 (0.04)		
	**TEMPO**	0.23 (0.02)		
	**SFF**	0.34 (0.03)		
**Surface Signal Intensity (ppm)**			63.2	55.4
**Total Signal Intensity (ppm)**			68.1	55.5

Table S3. C4 integration regions of unmodified and modified fibers

<table>
<tr><td colspan="3">C4 integration regions normalized to 1.0</td><td>Before Oxidation(s)</td><td>After Oxidation(s)</td></tr>
<tr><td rowspan="3">Relative Surface Signal Intensities</td><td>REF</td><td>0.47 (0.05)</td><td rowspan="3"></td><td rowspan="3"></td></tr>
<tr><td>TEMPO</td><td>0.44 (0.04)</td></tr>
<tr><td>SFF</td><td>0.44 (0.04)</td></tr>
<tr><td colspan="3">Surface Signal Intensity (ppm)</td><td>86.2</td><td>78.9</td></tr>
<tr><td colspan="3">Total Signal Intensity (ppm)</td><td>94.0</td><td>78.9</td></tr>
</table>

Table S4. Carbonyl integration regions of unmodified and modified fibers

<table>
<tr><td colspan="3">Carbonyl integration regions normalized to 6.0</td><td>Before Oxidation(s)</td><td>After Oxidation(s)</td></tr>
<tr><td rowspan="3">Relative Carbonyl Signal Intensities</td><td>REF</td><td>N/A</td><td rowspan="3"></td><td rowspan="3"></td></tr>
<tr><td>TEMPO</td><td>0.096 (0.01)</td></tr>
<tr><td>SFF</td><td>0.074 (0.01)</td></tr>
<tr><td colspan="3">Carbonyl Signal Intensity (ppm)</td><td>180.0</td><td>160.0</td></tr>
<tr><td colspan="3">Total Cellulose Intensity (ppm)</td><td>120.0</td><td>40.0</td></tr>
</table>

Supplementary Smart Filter Information

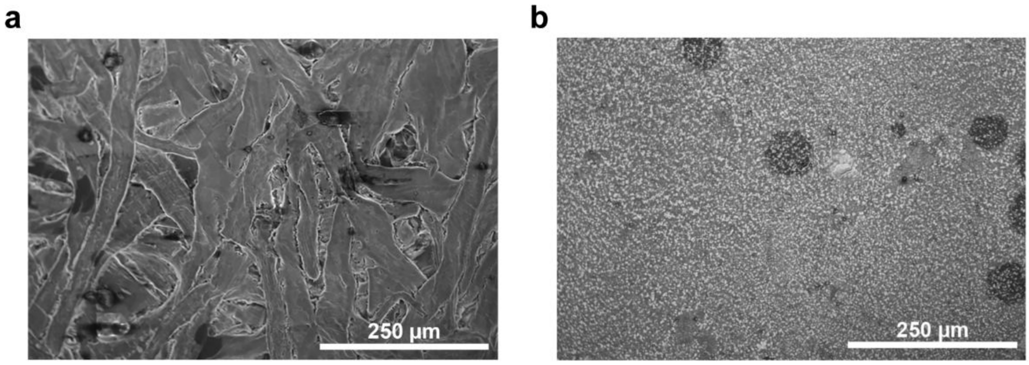

Figure S2. FE-SEM micrographs of the area used for EDS mapping for a) unfibrillated and b) fibrillated SFF filter.

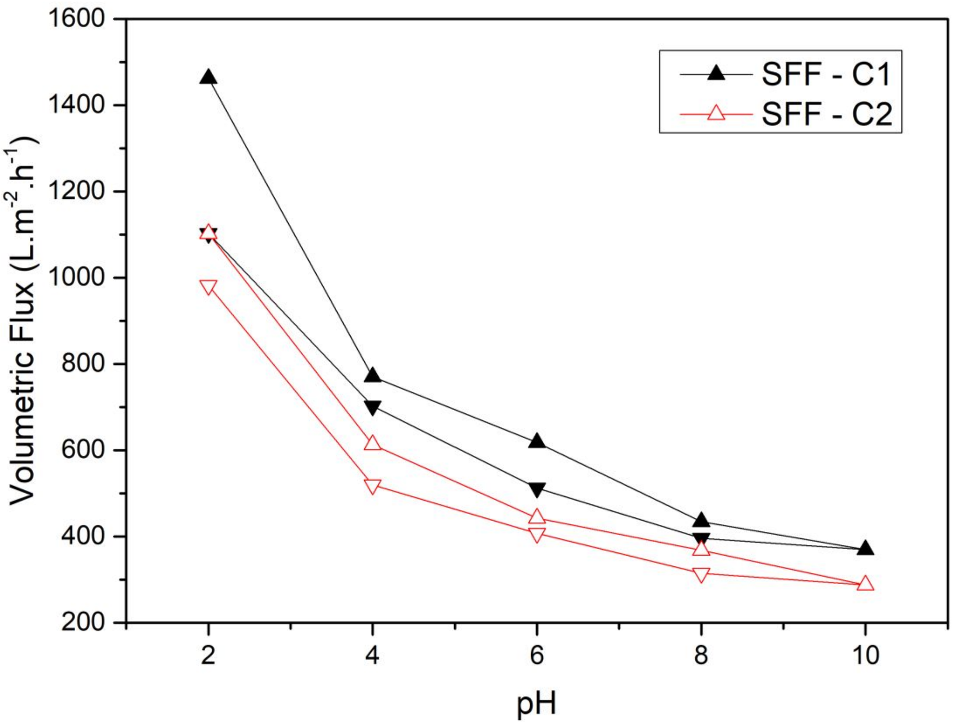

Figure S3. Volumetric flux of water through the SFF smart filter at different pH values where the pH was cycled between 2 and 10. First cycle is shown in black and the second cycle is shown in red. Triangle represents increasing pH in the cycle and inverted triangle represents decreasing pH in the same cycle.

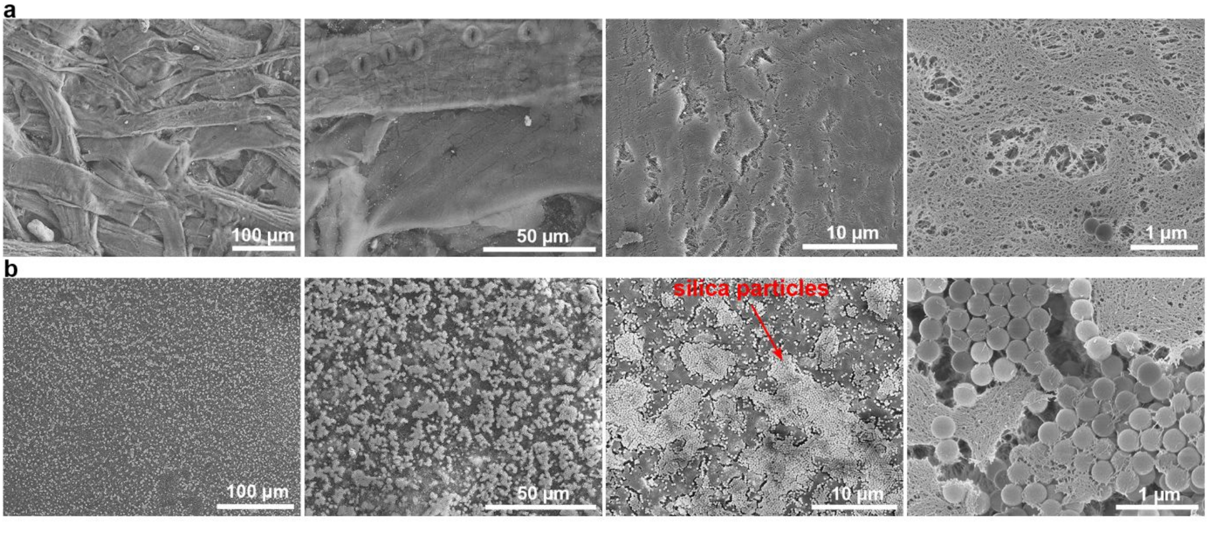

Figure S4. FE-SEM micrographs of the a) unfibrillated and b) fibrillated filter at different magnifications with apparent silica particles.

Computation of the Swelling Potential Using Donnan Theory

If the pH_g is known, the degree of dissociation (α) of the carboxyl groups inside the fiber wall can be calculated according to the following relationship

$$pH_g = pK_a + log_{10}\left(\frac{\alpha}{1-\alpha}\right) \quad \text{(S1)}$$

Where pK_α for the carboxyl groups was taken as 4.0 in this study.

Due to the Gibbs-Donnan effect resulting from the presence of immobile carboxyl groups inside the fiber wall, the ions inside the fiber gel (pH_g) and in the solution (pH_s) are distributed unevenly. Using the Donnan equilibrium, the distribution of the different mobile ions (λ) can be written as follows

$$\lambda = \frac{[H_3O^+]_g}{[H_3O^+]_s} = \frac{[Na^+]_g}{[Na^+]_s} = \frac{[OH^-]_s}{[OH^-]_g} = \frac{[Cl^-]_s}{[Cl^-]_g} \quad \text{(S2)}$$

Considering that the distribution of sodium ions depends on the concentration of all other ions present in the system, the concentration of sodium ions inside and outside (in the solution) fiber wall can be expressed as the following, respectively

$$[Na^+]_g = [OH^-]_g + [Cl^-]_g + [COO^-]_g - [H_3O^+]_g \quad \text{(S3)}$$

$$[Na^+]_s = [OH^-]_s + [Cl^-]_s - [H_3O^+]_s \quad \text{(S4)}$$

$[COO^-]_g$ (or cα, where c is the concentration of carboxyl groups) represents the concentration of dissociated carboxyl groups in the gel

The autoprotolysis of water can be shown as the following, where K_W is the autoprotolysis constant of water

$$K_w = [H_3O^+][OH^-] \quad \text{(S5)}$$

By rearranging the third term in equation S2

$$\frac{[OH^-]_s}{[OH^-]_g} = \frac{\frac{K_w}{[H_3O^+]_s}}{\frac{K_w}{[H_3O^+]_g}} \quad \text{(S6)}$$

Combining the equations S3-S6 with equation S2 gives

$$\lambda = \frac{[H_3O^+]_g}{[H_3O^+]_s} = \frac{\frac{K_w}{[H_3O^+]_g} + [Cl^-]_g + c * \alpha - [H_3O^+]_g}{\frac{K_w}{[H_3O^+]_s} + [Cl^-]_s - [H_3O^+]_s} = \frac{\frac{K_w}{[H_3O^+]_s}}{\frac{K_w}{[H_3O^+]_g}} = \frac{[Cl^-]_s}{[Cl^-]_g} \quad \text{(S7)}$$

Further rearrangement of the above equation yields the following expression, which describes the distribution of ions

$$\lambda = \sqrt{1 + \left(\frac{c\alpha}{\frac{K_w}{[H_3O^+]_g} + [Cl^-]_g} \right)} \quad \text{(S8)}$$

The swelling potential, or E (mol/L), is caused by the uneven distribution of ions and it represents the excess of ions inside the fiber wall as the degree of dissociation increases

$$E = [H_3O^+]_g + [OH^-]_g + [Na^+]_g + [Cl^-]_g - [H_3O^+]_s - [OH^-]_s - [Na^+]_s - [Cl^-]_s \quad \text{(S9)}$$

Using the distribution, S8 and S9, the following expression for E can be obtained

$$E = c\alpha + 2(\frac{K_w}{[H_3O^+]_g} + [Cl^-]_g)(1 - \lambda) \quad \text{(S10)}$$

Which can be simplified as follows

$$E = \left(\frac{\lambda - 1}{\lambda + 1} \right) c\alpha \quad \text{(S11)}$$

Calculation of Osmotic Pressure inside the Fiber Wall

Osmotic pressure (Π) inside the fiber wall was calculated using the classical equation of van't Hoff. The osmotic pressure is given by the following expression,

$$\Pi = \left(\frac{n}{V} \right) RT \quad \text{(S12)}$$

where V is the volume of pure solvent (water) inside the fiber wall, n is the concentration of dissociated ions, R is the universal gas constant and T is the absolute temperature.
